# Micro 3D printing of a functional MEMS accelerometer

**DOI:** 10.1038/s41378-022-00440-9

**Published:** 2022-09-19

**Authors:** Simone Pagliano, David E. Marschner, Damien Maillard, Nils Ehrmann, Göran Stemme, Stefan Braun, Luis Guillermo Villanueva, Frank Niklaus

**Affiliations:** 1 KTH Royal Institute of Technology, Division of Micro and Nanosystems, Malvinas väg 10, Stockholm, Sweden; 2 École Polytechnique Fédérale de Lausanne (EPFL), Advanced NEMS Laboratory, Institute of Mechanical Engineering, 1015 Lausanne, Switzerland; 3 Hochschule Kaiserslautern, University of Applied Sciences, Informatik und Mikrosystemtechnik, Campus Zweibrücken, Germany

**Keywords:** Electrical and electronic engineering, Nanoscience and technology

## Abstract

Microelectromechanical system (MEMS) devices, such as accelerometers, are widely used across industries, including the automotive, consumer electronics, and medical industries. MEMS are efficiently produced at very high volumes using large-scale semiconductor manufacturing techniques. However, these techniques are not viable for the cost-efficient manufacturing of specialized MEMS devices at low- and medium-scale volumes. Thus, applications that require custom-designed MEMS devices for markets with low- and medium-scale volumes of below 5000–10,000 components per year are extremely difficult to address efficiently. The 3D printing of MEMS devices could enable the efficient realization and production of MEMS devices at these low- and medium-scale volumes. However, current micro-3D printing technologies have limited capabilities for printing functional MEMS. Herein, we demonstrate a functional 3D-printed MEMS accelerometer using 3D printing by two-photon polymerization in combination with the deposition of a strain gauge transducer by metal evaporation. We characterized the responsivity, resonance frequency, and stability over time of the MEMS accelerometer. Our results demonstrate that the 3D printing of functional MEMS is a viable approach that could enable the efficient realization of a variety of custom-designed MEMS devices, addressing new application areas that are difficult or impossible to address using conventional MEMS manufacturing.

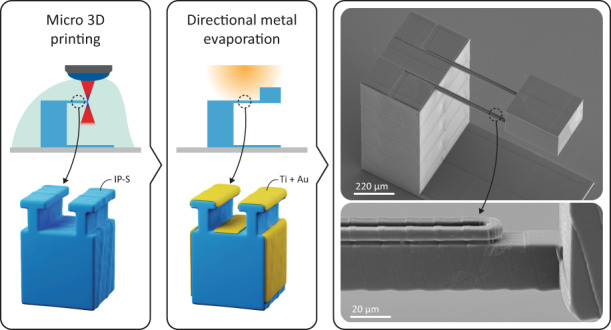

## Introduction

Microelectromechanical system (MEMS) sensors, including accelerometers, gyroscopes, pressure sensors, and microphones, have seen great success in recent decades. Today, they are ubiquitous in many applications, such as mobile phones, cars, gaming consoles, and navigation systems. However, the production of MEMS components in small and medium-sized batches for specialized high-value applications, such as robotics, aerospace, and medicine, is often hindered by the high start-up cost of manufacturing process development and device design optimizations. These start-up costs are fixed costs that do not scale with increasing production volumes^1^. Therefore, scalability is not just an advantage in MEMS production but a necessity to ensure a high return on investment. As a result, the development of novel commercial MEMS devices is often limited to devices that address very high-volume markets. For some application areas with small- and medium-sized volumes, this requires engineers to use the suboptimal MEMS devices available on the market. It may not even be possible to address other applications in an economically viable way. Emerging micro 3D printing technologies could fill this gap and enable the manufacturing of small- and medium-volume batches of MEMS components. This includes the rapid prototyping of MEMS toward highly specialized custom applications and the cost-efficient manufacturing of MEMS in small- to medium-sized manufacturing volumes, i.e., from a few hundred to a few thousand devices.

In recent decades, macroscale 3D printing techniques have been used extensively for the rapid prototyping of mechanical parts^2^ due to their flexibility and versatility. Recently, they have also been introduced in the manufacturing of final components in many sectors, such as automotive^3^ and avionics^4^, especially where complex component geometries and low manufacturing volumes are needed. More recently, different 3D printing techniques have been used to realize functional macro- and mesoscale sensor devices^5,6^. Among them, macroscale inertial sensors have been fabricated using fused filament fabrication^7,8^, laser powder bed fusion^9^, and stereolithography^10–12^. These devices have footprints of several mm^2^ up to several cm^2^, and therefore they are not suitable for applications where miniaturization is critical. The footprint reduction of these types of 3D-printed sensor devices remains challenging because of the intrinsic limitations of the used 3D printing techniques, which can at best achieve dimensions as small as tens or hundreds of micrometers^5,6^, thereby setting a practical limit to the miniaturization and precision of the sensors, along with the related bandwidth limitations.

Among the 3D printing techniques suitable for realizing microscale devices, two-photon polymerization, also referred to as multiphoton polymerization or direct laser writing, is well suited for printing MEMS devices. 3D printing by two-photon polymerization offers resolutions of below 1 µm in all spatial directions^6,13^, matching and in some cases overcoming the resolution of cleanroom-based lithographic processes. Such a high resolution is critical in the realization of MEMS devices; hence, this technology has been used to realize microfluidic circuits^14^, optical devices^15^, and scaffolds for tissue engineering^16^. In addition to being capable of micro- and nanoscale printing, two-photon polymerization also allows 3D printing with a high degree of design freedom in 3D space. However, the realization of electrically functional transducers and sensor structures at the microscale is very challenging with this technique. While 3D printing by two-photon polymerization with subsequent Al sputtering has been used to fabricate thermomechanical and electrostatic actuators^17,18^, 3D-printed microscale inertial sensors, such as MEMS accelerometers and gyroscopes, have not yet been realized. In the present work, we demonstrate the first 3D-printed functional MEMS accelerometer using two-photon polymerization in combination with metal evaporation to form strain gauge transducers. We characterized the 3D-printed MEMS accelerometer and confirmed its successful operation.

## Results

### 3D-printed MEMS accelerometer

**Fig. 1:**
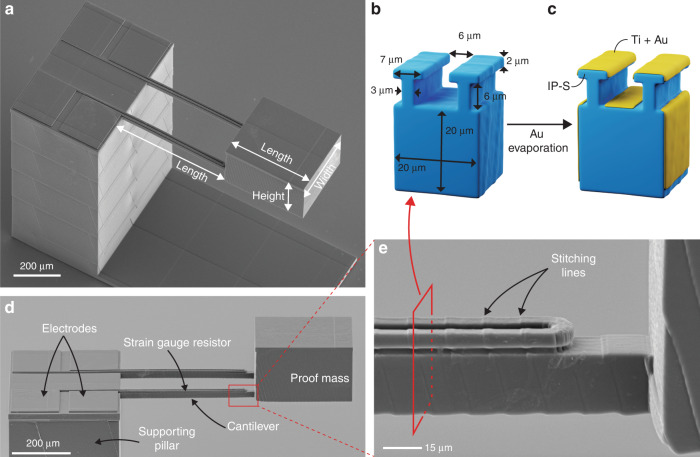
3D-printed accelerometer. **a** SEM image of the 3D-printed accelerometer structure. **b** 3D schematic view of the cantilever cross-section before metal evaporation. **c** 3D schematic view of the cantilever cross-section after metal evaporation showing the shadow masking mechanism that enables the electrical isolation of the resistors. **d** SEM image of a lateral view of the top part of the device. **e** Close-up view of the T-shaped resistors on top of the cantilevers. The structures shown in the SEM images were coated with a thin sputtered Au–Pt layer to improve the SEM image quality.

To demonstrate the practical feasibility of 3D-printed functional MEMS accelerometers, we designed an accelerometer structure that could be 3D printed by two-photon polymerization, with a subsequent directional metal deposition step for forming the strain gauge transducer elements, electrical interconnects, and probing electrodes (Fig. 1a). We printed the accelerometer structure on a glass substrate using a Nanoscribe Photonic Professional GT2 (Nanoscribe GmbH, Germany) 3D printer and commercial IP-S resin (Nanoscribe Gmbh, Germany). The mechanical accelerometer structure consisted of a supporting pillar with two single-sided clamped horizontal cantilevers and a proof mass attached at the end of the two cantilevers (Fig. 1a and Supplementary Information, Fig. S1). The design freedom offered by the 3D printing process allowed us to pattern shadow-masking structures with T-shaped cross-sections (Fig. 1b) on top of the cantilevers and the supporting pillar to define the areas of the strain gauge transducers, the electrical interconnects and the probing electrodes (Fig. 1d, e). The T-shaped shadow-masking structures, in combination with the deposition of a 10 nm-thick layer of Ti and a 30 nm-thick layer of Au using a directional evaporation process (Fig. 1c, see the section “Materials and methods”), resulted in the formation of electrically separated metal coatings on the different surfaces of the 3D-printed accelerometer structure, thereby forming electrically isolated probing electrodes, interconnects, and resistors acting as metal strain gauges. We chose the dimensions of the T-shaped structures (Fig. 1b) to ensure both (a) reliable 3D printing of the structures using the chosen resin and microscope objective and (b) effective shadow masking during metal deposition resulting in electrically disconnected metal lines and coatings. The top surfaces of the supporting pillar and the cantilevers were placed at the same height to allow the simple formation of the electrical interconnects between the strain gauge resistors and the probing electrodes (Fig. 1d). The bottom surface of the proof mass was leveled with the bottom surface of the cantilevers to avoid so-called “flying blocks” during printing, which are printing blocks that are not attached to a solid structure at the time of printing.

The operation principle of our MEMS accelerometer resembles that of a standard piezoresistive MEMS accelerometer, i.e., an external acceleration acting on the proof mass in the direction perpendicular to the substrate surface results in a force that causes the cantilevers to bend (according to Newton’s second law *F* = m*a with *F* = force, *m* = proof mass, and *a* = acceleration). The bending of the cantilevers results in the straining of the resistive metal strain gauges on top of the cantilevers and the associated change in the electrical resistance of the strain gauges. The resistance change of the strain gauge is correlated to the induced strain and ultimately to the applied acceleration.

For the layout of the accelerometer design, we developed a parametrized finite-element model in COMSOL^®^ to compute the geometrical parameters required to obtain a measurement range of the accelerometer of 1–10×*g*, which is a typical specification in consumer, navigation, or industrial applications^19^. Details of the modeling are described in the “Materials and methods” section. To ensure a high sensitivity of the mechanical accelerometer structure, i.e., a large bending displacement at low accelerations, the thickness, and width of the cantilevers must be minimized, while their length and the size of the proof mass must be maximized. Based on our prior experience with 3D printing long cantilever structures, we chose the cantilever thickness and width to be 20 µm each, allowing for the well-controlled printing of the cantilevers and of the two parallel T-shaped wire structures that form the strain gauge resistors on top of the cantilevers (Fig. 1d, e). Next, using the COMSOL® model, we performed a sweeping analysis of the remaining geometrical parameters, which were the length of the cantilevers and the mass of the attached proof mass, computed as the volume of the proof mass multiplied by the density of the polymer. Out of the parametric sweep, we selected a cantilever length of 500 µm and proof mass dimensions of 350 µm × 300 µm × 210 µm (length × width × height). For the 3D-printed accelerometer structures, we found that the actual length of the cantilevers consistently deviated from the designed length of 500 µm; hence, we adapted the dimensions of the COMSOL® model to the measured cantilever length of 480 µm.

### Accelerometer characterization

**Fig. 2:**
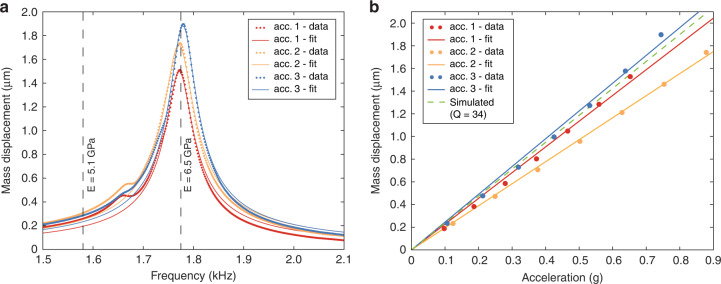
Mechanical characterization. **a** Amplitude of oscillation of the MEMS accelerometer proof mass at different frequencies using a piezoshaker driving voltage of 7 V. The resonance frequency was measured at the maximum oscillation amplitude between 1.77 and 1.78 kHz for all devices instead of 1.58 kHz, as predicted by the model developed in COMSOL^®^, using a Young´s modulus of 5.1 GPa as specified in the material datasheet^30^. The measured resonance frequency corresponds to an actual Young´s modulus of 6.5 GPa. Fitting to a Lorentzian curve yields *Q* factors of 36 (accelerometer 1), 31.7 (accelerometer 2), and 35.7 (accelerometer 3). **b** Measured and simulated amplitude of oscillation of the proof mass at the different accelerations applied by the piezoshaker. The amplitude was measured at the resonance frequency, and the simulated force was multiplied by the average *Q* factor of 34 to match the behavior at resonance.

To characterize the performance of the 3D-printed accelerometer, we fabricated three devices with identical designs and measured their resonance frequency, responsivity, and response stability over time. For this purpose, we used a setup consisting of a piezoshaker and a laser doppler vibrometer (LDV) that were both connected to a lock-in amplifier. The lock-in amplifier drove the piezoshaker at the desired frequency and simultaneously demodulated both the signal from the laser doppler vibrometer, which is correlated to the amplitude of the mechanical oscillation of the proof mass and the amplitude of the resistive strain gauge transducer at that same frequency. After we calibrated the piezoshaker (see the “Materials and methods” section), we characterized the mechanical response of the accelerometer at its resonance frequency. We evaluated the mechanical response by sweeping the frequency of the driving voltage between 1.4 and 2 kHz. This signal was sent to the piezoshaker with different amplitudes, ranging from 1 to 7 V_rms_. We measured the resonance frequency of the three accelerometers to be reliably within the range of 1.775 kHz ± 5 Hz, with *Q*-factors ranging between 31 and 36 (Fig. 2a, details in Supplementary Information, Section S6). We observed that, compared to the *Q*-factor of the same devices measured without contact probes (41.5 ± 1.4), the placement of the probes on the electrodes of the accelerometer influenced the *Q*-factor within a range of ±30%. At the same time, we did not observe any significant impact of the probes on the measured resonance frequency of the 3D-printed accelerometers. For each device and driving voltage amplitude, we plotted the maximum oscillation amplitude against the applied acceleration (Fig. 2b). The plots show a linear behavior of the mechanical response of the accelerometers within the range of evaluated vertical proof mass displacements of up to 1.9 µm. The displacement amplitude measurements were performed on top of the proof mass, and the exact location was chosen differently on each device to maximize laser reflection and minimize the noise in the laser doppler vibrometer read-out. We compared the measured resonance frequency and displacement amplitude to the simulated data from the adapted COMSOL^®^ model. The simulated resonance frequency was consistent with the measured resonance frequency of 1.775 kHz, assuming Young’s modulus of 6.5 GPa for the polymer. The displacement amplitude was extracted based on the average *Q*-factor of 34 at the center of the mass and plotted in Fig. 2b. The simulated data lie within the range of the different measured datasets, as expected. Furthermore, to obtain a basic understanding of the influence of the different design parameters on the mechanical behavior and the resonance frequency of the accelerometers, we developed two theoretical models based on Euler‒Bernoulli beam theory (see Supplementary Information, Section S5). With these models, we predicted resonance frequencies within 10% of the measured values using the elastic modulus extracted from our COMSOL^®^ simulation.

**Fig. 3:**
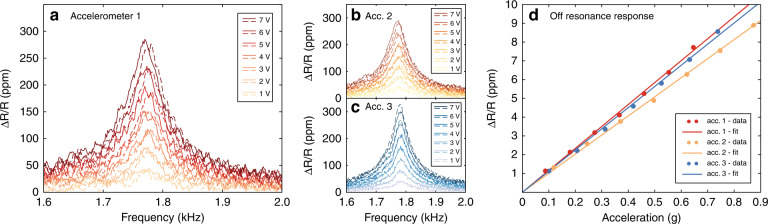
Electrical characterization. Relative resistance change (Δ*R*/*R*) measured at different frequencies and driving voltages of the piezoshaker on accelerometer 1 in (**a**), accelerometer 2 in (**b**), and accelerometer 3 in (**c**). The noticeable shift in resonance frequency is attributed to the increase in temperature in the polymer at large oscillation amplitudes. **d** Relative resistance changes of the strain gauge transducers as a function of the acceleration applied to the MEMS accelerometers computed off-resonance.

The responsivity of our 3D-printed accelerometer, defined as the relative resistance change (Δ*R*/*R*) as a function of the applied acceleration, was measured at the resonance frequency. Similar to our mechanical characterization, we swept the driving voltage from 1.4 to 2 kHz at voltages from 1 to 7 V_rms_. For each 3D-printed accelerometer and voltage, we carried out two frequency sweeps. From the downmixed voltage outputted by the lock-in amplifier for each sweep, we extracted the values of the relative resistance change of the strain gauge transducers and plotted them (Fig. 3a,b,c). From the maximum amplitude of the output signal of the lock-in amplifier, which occurred at the resonance frequency of the device, we computed the off resonance relative resistance change and plotted it against the corresponding acceleration applied by the piezoshaker for each device (Fig. 3d). Thus, we extracted responsivities ranging from 322 and 420 ppm/g at the resonance frequency for the three different devices, which, divided by the measured *Q*-factor of each device, yielded a responsivity of 11 ± 0.7 ppm/g at standard testing frequencies (100–160 Hz) for all three devices. Based on these values of responsivity and the measured mass displacement, we computed the gauge factor of our thin film strain gauge transducers to be within 3.5 ± 0.6 for all devices. The measured gauge factor was higher than that extracted from our adapted model simulated in COMSOL^®^, which yielded a value of 2, which is to be expected for bulk gold conductors but not thin film conductors (see the “Discussion” section).

**Fig. 4:**
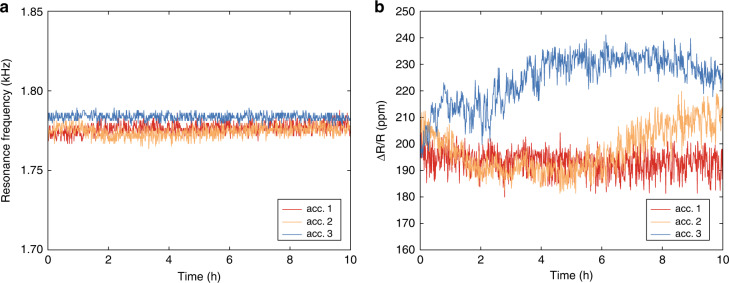
Stability evaluation. Measurements of relative resistance change and resonance frequency of the three devices over a period of 10 h using a constant piezoshaker driving voltage of 5 V_rms_. **a** For each device, the resonance frequency remained stable within an interval of ±3.8 Hz from the average resonance frequency. **b** Relative resistance change Δ*R*/*R*, computed at the resonance frequency, shifting by approximately 20 ppm with respect to the initial value of 205 ppm for both accelerometer 2 and 3, while remaining stable at 193 ± 5 ppm for accelerometer 1.

Important considerations for MEMS sensors utilizing polymers as structural materials are the sensing repeatability and long-term stability. To evaluate the stability of our 3D-printed MEMS accelerometers over time, we performed a 10-h measurement on each device. In these experiments, we continuously applied frequency sweeps at a constant driving voltage of 5 V_rms_ to the piezoshaker while tracking the relative resistance change of the strain gauge transducers. For each sweep, we extracted both the resonance frequency of the accelerometer and the maximum relative resistance change and plotted their behavior over time (Fig. 4). The measured resonance frequencies do not show significant drift over time throughout the measurements, remaining within a range of ±4 Hz from the average value for each device (Fig. 4a). This confirms that the mechanical response of our accelerometers over time is stable, without degradation of the mechanical properties of the polymer. Additionally, the relative resistance change of the strain gauge transducers did not shift significantly for the three devices during the duration of the measurements (Fig. 4b). For two of the devices, we observed a variation in the relative resistance change of approximately 20 ppm in the rolling average over 20 min of measurement, while the third device maintained a value of ±4 ppm from the initial value. These shifts in responsivity could be due to variations in the acceleration applied to the device, which has been measured to be within a similar range (see the “Materials and methods” section), and/or due to variations in the relative resistance change of the thin metal films deposited on the polymer that are subjected to mechanical loading and electrical current. The latter effect has been previously reported and is mostly attributed to the local cracking of the brittle adhesion layer^20^ and/or the rearrangement of grains in the polycrystalline metal films^21^. Temperature and relative humidity were monitored during these experiments to evaluate the impact of environmental parameters on the device performance in our experimental setup. Both of these environmental parameters showed minimal variations that did not contribute significantly to variations in the performance of the devices (see Supplementary Information, Section S7).

## Discussion

Here, we have demonstrated the manufacturing of three fully functional MEMS accelerometers using micro-3D printing and a subsequent simple directional metal evaporation step. The 3D printing of one accelerometer structure, including development and UV flood exposure, took 1 h 45 min (not optimized to achieve the shortest printing time), and the metal evaporation took 40 min, of which 30 min was due to the vacuum pumping of our equipment. Thus, the total time it takes to manufacture a single MEMS accelerometer is below 2 and a half hours, requiring only two relatively inexpensive commercial manufacturing tools (a two-photon polymerization 3D printer and a metal evaporator) and no cleanroom. Additionally, when fabricating a batch of several devices, some of the processes, such as UV exposure and metal deposition, can be carried out in parallel on many devices, thus considerably reducing the fabrication time required for each accelerometer. Furthermore, while two-photon polymerization 3D printers are still relatively expensive to acquire and operate compared to other 3D printing technologies, the productivity of these types of 3D printers is expected to increase in the next few years, as research on parallel beams^13^ and more sensitive photopolymers^22^ has shown promising results. Alternative 3D printing techniques that offer sub-µm resolution^23–25^ are more limited than two-photon polymerization in terms of geometry and printing speed^13^. Our demonstration suggests that this approach is feasible for prototyping MEMS devices and manufacturing small- and medium-sized batches of tens to a few thousand MEMS sensors per year in an economically viable way. This is something that has not been possible until now because the start-up costs for manufacturing a MEMS product using conventional semiconductor technology are on the order of hundreds of thousands of USD and the lead times are several months or more. The new capabilities offered by 3D-printed MEMS could result in a paradigm shift in MEMS and sensor manufacturing, making MEMS innovation capabilities available to a broader community and making it possible to efficiently address specialty applications and niche markets with custom-designed MEMS products. Furthermore, the increased design freedom that 3D printing offers over the conventional silicon surface and bulk micromachining technologies enable the realization of structural complexities that have not been possible before. In this work, we chose a comparably straightforward design of accelerometer cantilevers with a squared cross-section to show the simplicity and ease of use of this new manufacturing approach. However, the 3D printing capabilities of two-photon polymerization allow more advanced sensor designs, such as those with varying cross-sections^26^ or cellular microarchitectures^27^, which will have a significant impact on the device performance and functionality and will ultimately result in new and improved MEMS devices.

Our demonstration of a 3D-printed MEMS accelerometer involves a polymer being used as the structural material. While most MEMS are made of silicon due to their excellent mechanical properties, polymers are increasingly being explored as MEMS and sensor materials, including for flexible and biodegradable devices^28^. One important concern regarding the use of polymers as structural MEMS materials is the repeatability of their performance and stability over time^29^. Furthermore, the mechanical properties of polymers, such as the elastic modulus and tensile strength, can be affected by environmental conditions such as temperature and humidity, although this can be mitigated by device packaging. We have demonstrated the mechanical stability of our polymer MEMS accelerometer by measuring its resonance frequency over more than 800 frequency sweeps over a period of 10 h, which consisted of more than 60 million oscillations. We found that the resonance frequency and thus the mechanical and dimensional properties of the polymer structure remained stable throughout these experiments. The measured resonance frequency (1.775 kHz) was in agreement with the one predicted by the simulation model, taking into account an actual measured length of the 3D-printed cantilevers of 480 µm (the nominal length was 500 µm) and assuming Young’s modulus of the printed IP-S polymer of 6.5 GPa (the value indicated in the material data sheet^30^ is 5.1 GPa) and a density of 1100 kg/m^3^ (from the material data sheet^30^). The length of the suspended cantilevers was approximately 4% shorter than that of the nominal design (Supplementary Information, Section S4). This level of shrinkage is within the typical expected range of the IP-S polymer^29^. Post-printing shrinkage is a known phenomenon in two-photon polymerization that affects polymeric resins based on acrylates, such as IP-S, but it is a consistent effect that can be mitigated by using shrinkage compensation schemes^31^. The adjusted Young’s modulus value is within a tolerance that can be expected, as it is known that the mechanical properties of two-photon polymerized materials can depend on the printing parameters, such as laser pulse energy, printing speed, and overlap between laser pulses^32^. An increase in stiffness of two-photon cross-linked polymer materials has been previously demonstrated to occur with higher levels of cross-linking^33^, which was also the case for our 3D-printed accelerometer structures due to the UV flood exposure performed after the development and the high number of stitching regions at the interface between adjacent printing blocks along the cantilever length that received a double exposure dose. A potential advantage of using polymers as structural materials in MEMS is their very low elastic moduli, which allow very large displacements of proof masses to be achieved even at low accelerations and with relatively short cantilevers, which could enable limits of detection as low as 1 µg/$\sqrt {{{{\mathrm{Hz}}}}}$ in devices with a small footprint^34^. In the future, 3D-printed composite materials that can be sintered into nonpolymeric structures after printing^35,36^ may be employed as structural materials for 3D-printed MEMS to avoid the disadvantages of polymers, although the sintering step typically induces large internal stresses that can cause deformation of the printed structures.

In our 3D-printed MEMS accelerometer, we used a 40 nm-thick metal layer for the resistors of the strain gauge transducers. In accelerometers using strain gauges, the accelerometer responsivity depends on its capability to turn acceleration into the straining of the resistor and on the capability of the resistor to transduce strain into a resistance change. The straining of the resistors is induced by the bending of the cantilevers resulting from an acceleration acting on the proof mass. The cantilever bending depends on the cantilever geometry and material. As described by our theoretical model (Supplementary Information, Section S5), cantilever and proof mass displacements are directly proportional to the cube of the cantilever length and inversely proportional to the second moment of area of the cantilever cross-section and to the elastic modulus of the cantilever material. Cantilever bending induces stresses and strains varying across the vertical cross-section of the cantilever, which is at maximums at the bottom and top surfaces of the cantilever, where the strain gauge resistors are positioned to take full advantage of the cantilever bending. The strain induced in the resistors because of cantilever bending can be computed using an analytical model^37^ (see Supplementary Information, Section S5). The ratio between the relative resistance change Δ*R*/*R* and the strain is often called the gauge factor, and it is the sum of the geometric changes of the resistor and the change in the resistivity of the strain gauge material due to the applied stress. In metals, this change in resistivity is often negligible, and the gauge factor is usually only a result of the change in the geometry of the metal line. However, a slightly higher gauge factor has been reported for thin films of Au, with thicknesses of 30 nm or below^38^, where the additional change in resistivity is attributed to both surface roughness and the presence of boundaries between grains in the polycrystalline metal films^39^. We estimated the gauge factor of our thin-film Au transducers to be 3.5 ± 0.6 for the three devices by measuring the relative resistance change Δ*R*/*R* of the transducers, as discussed in the “Results” section, and by calculating the strain with theoretical formulas^37^ using the measured mass displacement amplitude, indicating that there are contributions from both the strain gauge deformation and the resistivity change. The measured responsivity of 11 ppm/g of our accelerometers is within the range of other published accelerometers^40^ and commercially available accelerometers^41^, for example, those used for shock testing. A larger responsivity can in principle be achieved with our accelerometer designs by adjusting the geometrical parameters through a trade-off with a lower resonance frequency. A larger responsivity could also be achieved without lowering the resonance frequency, either by implementing optimized accelerometer designs^42^ or using resistors made of piezoresistive materials with larger gauge factors. Interesting alternatives to metals for resistive strain gauges could be evaporated germanium^43^ and sputtered amorphous carbon^44,45^, which have already been used as strain gauges on flexible substrates and feature gauge factors above 30. However, it is important that the temperatures involved in the material deposition are compatible with the 3D-printed polymer, which are typically below 300 °C.

To characterize the responsivity of our accelerometer, we performed measurements at resonance frequency to ensure that the output signal was significantly higher than the output noise, even at applied accelerations that were as low as 0.1 g. When no signal was applied to the piezoshaker, we measured a noise density of 90 nV/√Hz, which corresponds to noise equivalent accelerations of approximately 4 mg/√Hz at resonance and 0.2 g/√Hz at standard testing frequencies (100–160 Hz). The noise density of the output signal can be decreased to 4 nV/√Hz with further optimization of the read-out circuit or even lower by using special low-noise application-specific integrated circuits (ASIC) for piezoresistive accelerometers^34^. Such low noise power densities would result in a noise equivalent acceleration in the order of 10 mg/√Hz at standard testing frequencies, which is comparable to those of commercially available MEMS accelerometers^19^. Furthermore, when operating the accelerometer at the resonance frequency, we achieved large proof mass displacements at accelerations of 0.9×*g*. In this way, we have demonstrated the linearity of the accelerometer response at proof mass displacements of up to 1.9 µm. This corresponds to cantilever deflections of approximately 1.2 µm at the end of the cantilevers and would occur only at accelerations >35×*g* at standard testing frequencies.

Taken together, our results demonstrate that functional 3D-printed MEMS accelerometers are practically possible and hold promise for competitive performance. Our proposed approach for the additive manufacturing of MEMS has the potential to be applied to a variety of MEMS sensors, such as pressure sensors, microphones, gyroscopes, and flow sensors. Moreover, 3D printing will enable innovative and complex device geometries for novel MEMS sensors that are not currently possible to realize using conventional 2.5D silicon micromachining. The strategy we use to selectively functionalize the surfaces of the 3D-printed MEMS structure by integrating shadow-masking elements in combination with directional material deposition is versatile and facilitates innovative designs and the integration of a variety of transducer elements, such as piezoresistors, piezoelectric elements, and nanowire elements. Importantly, the quick turn-around between the design and the fabrication of small batches of 3D-printed MEMS allows us to assess the performances of the devices and optimize them in a matter of a few hours. From an industrial perspective, this dramatically reduces the start-up cost for manufacturing novel custom MEMS devices for small- and medium-volume applications, the fabrication cost of which would be prohibitive using standard microfabrication techniques. Thus, our approach to the additive manufacturing of functional MEMS, together with the wide range of promising innovations the technology enables, facilitates a completely new 3D design and manufacturing paradigm for MEMS, which holds great promise for future research and applications in important fields such as robotics, aerospace, and medicine.

## Materials and methods

### Fabrication of the 3D-printed MEMS accelerometer

The MEMS accelerometer structure was 3D printed by two-photon polymerization using a Nanoscribe Photonic Professional GT2 tool (Nanoscribe GmbH, Germany). Therefore, the negative photoresist IP-S (Nanoscribe Gmbh, Germany) was used in combination with the Dip-In Laser Lithography printing mode. The printing substrate was an indium tin oxide-coated glass substrate. The printing was performed using a ×25/NA 0.8 objective lens (Carl Zeiss AG, Germany) at a laser power of 50 mW with a scan speed of 100 mm/s and slicing and hatching distances of 1 and 0.5 μm, respectively (see Supplementary Information, Section S2). For the printing, the accelerometer structure was defined by several printing blocks that were stitched together. Some of the blocks were printed in “Solid” printing mode, while others were printed in “Shell & Scaffold” printing mode. In Solid mode, the laser scans the entire printed volumes, while in Shell & Scaffold, the laser scans only the outer shell of the volume and a supporting scaffold inside of the volume. The Shell & Scaffold printing mode is much faster than the Solid printing mode, but it achieves lower resolutions. The large supporting pillar was printed using the Shell & Scaffold printing mode. The electrodes, wires, and free-hanging elements, i.e., the cantilevers and proof mass, were printed instead using the Solid printing mode to ensure the highest printing quality (Supplementary Information, Fig. S3). The printing file was generated with the Describe software (Describe 2.5.5, Nanoscribe GmbH, Germany). At first, two print job files were generated from the same 3D CAD file (.stl), one for each printing mode (Shell & Scaffold mode, and Solid mode), ensuring that stitching lines would perfectly match. Then, the two files were merged into a single print job file, where the blocks of the cantilevers and the mass were deleted from the Shell & Scaffold file and replaced by those in the Solid file.

After 3D printing, the structures were developed in propylene glycol methyl ether acetate (PGMEA) for 20 min and in isopropyl alcohol (IPA) for 5 min while holding the glass slide in the vertical position. Then, the 3D-printed structures were dried in the air at room temperature. After development, the structures were exposed to a UV flood using an LED exposure unit for 5 min (12 mW/cm^2^ @ 365 nm) to cross-link all the internal volumes of the structure that were printed in the Shell & Scaffold printing mode and thus were not crosslinked by laser exposure. Next, the top surfaces of the 3D-printed structure were coated with a 10 nm-thick layer of Ti and a 30 nm-thick layer of Au using directional e-beam evaporation (Provac PAK 600 Coating System) in the direction perpendicular to the surface of the glass substrate. The three devices were coated simultaneously, with the rotation speed of the planetary set to zero and the sample holder carefully placed above the crucible. The thickness of the stack of metal was measured with a mechanical profilometer to be 40 ± 2 nm, while the Ti thickness was measured to be 10 ± 1 nm after Au wet etching. After metal deposition, the finished MEMS accelerometers were inspected by SEM (FEI Nova 200 Dual Beam, FEI Company Inc., USA).

### Simulation model of the MEMS accelerometer

The finite element method (FEM) in the COMSOL^®^ software (COMSOL Multiphysics ^®^ v. 5.6, COMSOL AB, Sweden) was used to create a simulation model of the accelerometer structure. A tetrahedral mesh for the larger volumes of the accelerometer structure and a quadratic mesh for the cantilevers and the resistors were used to decrease the number of mesh points in the smaller device features without losing accuracy. In the initial model used to design the structure, for the cured IP-S polymer, Young’s modulus of 5.1 GPa and a density of 1100 kg/m^3^ ^30^ were used. The model was employed to estimate the length of the cantilever and the size of the proof mass required to achieve the target specification of the accelerometer prior to device fabrication. Therefore, the physics module “Structural Mechanics” and the “Stationary” solver were used to carry out a parametric sweep analysis of the cantilever length and the proof mass size. To minimize the computation time, only half of the structure was simulated, cut along the direction of the cantilever, and the boundary condition “Symmetry” was applied to the cross-sectional surface. After an initial assessment of the printed structure, an adapted model in COMSOL^®^, with shortened cantilevers (480 µm) and adjusted Young´s modulus (5.9 GPa) was created. The simulated data extracted from this model were compared to the measured data. The model was also used to compute the resistance change of the thin stacked metal films forming the strain gauges (Ti and Au). Therefore, the resistivity of Au was set to 52 nΩ*m, which was extracted by a 4-probe sheet resistance measurement performed on a 3D-printed test structure that was coated with the same metal stack used in the accelerometer strain gauge. The resistivity of Ti was measured to be 545 nΩ*m with the same method and set in the COMSOL^®^ model, but being considerably larger than that of Au, it is not expected to contribute significantly to the electrical conduction. We added the “Electric Currents” module in the COMSOL^®^ model and applied a sweep of accelerations ranging from 0.2 × *g* to 1 × *g* acting on the entire structure, multiplied by the *Q* factor to simulate the proof mass and cantilever displacement at resonance. The calculated resistance change of the thin stacked metal films was consistently lower than the measured values, which may be because the COMSOL^®^ model takes into account only the geometrical deformation of the resistor. Using the model, the strain applied to the cantilever and the metal strain gauge was calculated, along with its relative resistance change. Based on this, the gauge factor was calculated to be equal to 2 using the COMSOL^®^ model, which corresponds to the expected gauge factor of a Ti/Au stack in which the resistance change is purely geometrical, instead of the actual gauge factor of 3.5 that we measured in our accelerometer.

To estimate the cross-responsivities of our devices for the acceleration components along the *x*- and *y*-axes, we used our COMSOL® model and computed cross-responsivities of 1.5% and 22% along the *y*-axis (perpendicular to the cantilevers) and *x*-axis (parallel to the cantilevers), respectively. Accelerations along the *y*-axis induce a rotation of the mass that induces opposite bending of the two cantilevers, resulting in opposite resistance changes. Thus, the impact of *y*-axis accelerations on the *z*-axis measurements can be further minimized by appropriately arranging the two resistors in a Wheatstone bridge configuration. Accelerations along the *x*-axis induce a mass rotation analogous to that induced by *z*-axis accelerations. The influence of this type of acceleration can be minimized by implementing an accelerometer design in which the neutral axis of the cantilevers is aligned with the center of the proof mass (Fig. S8.1, Supplementary Information). In this way, proof mass rotations due to *x*-axis acceleration are minimized. Our simulations show that this can decrease the cross-sensitivity along the x-axis to 0.3% without affecting the responsivity along the *z*-axis (see Supplementary Information, Section S8). To demonstrate that such a design can be fabricated, we implemented and 3D-printed the refined accelerometer structure (Fig. S8.2, Supplementary Information).

### Characterization of the accelerometer

**Fig. 5:**
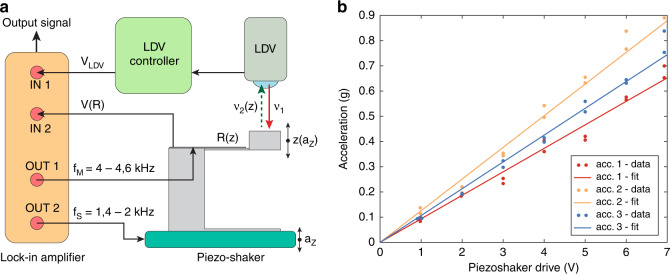
Measurement setup and piezoshaker calibration. **a** Schematic of the setup used to measure the responsivity of the 3D-printed MEMS accelerometer. The lock-in amplifier was used to drive the piezo-shaker, extract the signal from the resistor through downmixing and demodulate the laser doppler vibrometer signal. **b** Acceleration applied by the piezoshaker was measured with the laser doppler vibrometer on top of the supporting pillars of the three devices under different driving voltages at a frequency of 1.775 kHz. The corresponding linear fits are shown. A variation of about ±10% between the different devices can be seen.

The measurement setup consisted of a piezoshaker (TA0505D024, Thorlabs, USA), a lock-in amplifier (H2FLI 50 MHz, Zurich Instruments, Switzerland), and a laser doppler vibrometer (OFV-551, Polytec, Germany) with the controller (OFV-5000, Polytec, Germany), as shown in Fig. 5a. For calibration, the mechanical response of the piezoshaker to the electrical stimulation was measured with the laser doppler vibrometer. First, the 3D-printed MEMS accelerometers were mounted on the piezoshaker together with the glass slide they were printed on using double-sided tape. Then, the mechanical actuation provided by the piezoshaker to the accelerometers was characterized. The laser doppler vibrometer measured the oscillation velocity of the top surface of the supporting pillar of the accelerometers, from which the acceleration could be calculated by multiplying it with the angular frequency. The piezoshaker driving frequency was swept between 1.4 and 2 kHz, with voltage amplitudes ranging from 1 to 7 V_rms_. The frequency and amplitude of the mechanical vibration of the piezoshaker were set by the frequency and amplitude of the driving signal. This characterization was repeated for each accelerometer (1) directly after mounting each accelerometer device onto the piezoshaker and (2) after the stability measurement of the accelerometer device was completed. Figure 5b shows the consistent linear behavior of the average acceleration applied to each of the accelerometers with the amplitude of the driving voltage at its specific resonance frequency. The piezoshaker could generate accelerations of up to 0.9 × *g* (8.8 m/s^2^). We observed variations of applied accelerations of a maximum of 20% between different devices, but only a maximum variation of 8% for each single device. This observation suggests that the coupling efficiency of the double-sided tape might have a dependency on the mounting procedure and influence the applied accelerations. However, performing the calibration on the top surface of the supporting pillar instead of on the piezoshaker surface allowed us to compare the mechanical and electrical response of the accelerometer with the mechanical stimuli directly applied at the cantilever connection, avoiding any influence of the double-sided tape on the computed responsivity of the device. After the characterization of the piezoshaker, the probing electrodes of the accelerometer were contacted with probes and thus electrically connected to the measurement setup. The lock-in amplifier generated both the sinusoidal signal driving the piezoshaker and the sinusoidal signal applied to the strain gauge transducers of the accelerometers. The piezoshaker was actuated with the same frequency sweeps and voltages as during the calibration. At the same time, the signal applied to the strain gauge transducers was swept in a frequency range between 4 and 4.6 kHz. The multiplication of that sinusoidal signal with the resistance change (also sinusoidal) created signals at two new frequencies located at both the addition and the subtraction of the original signals: in our case, at 2.6 and 5.4 kHz. Demodulating the signal at the downmixed frequency (2.6 kHz) yields a voltage component proportional to the resistance change in the sensor.

## Supplementary information

Supplementary Information_Micro 3D printing of a functional MEMS accelerometer
