# Supplementary material for: Micro 3D printing of a functional MEMS accelerometer
Source: Microsyst Nanoeng. 2022 Sep 19;8:105. doi: 10.1038/s41378-022-00440-9 (PMC9482918; doi:10.1038/s41378-022-00440-9)
Supplement: Supplementary file 1 — Supplementary Information_Micro 3D printing of a functional MEMS accelerometer [file 41378_2022_440_MOESM1_ESM.docx]

Supplementary Information

Micro 3D Printing of a Functional MEMS Accelerometer

Simone Pagliano*, David E. Marschner*, Damien Maillard, Nils Ehrmann, Göran Stemme, Stefan Braun, Luis Guillermo Villanueva and Frank Niklaus

**S1. Accelerometer Design**

Drawings of the design of the 3D printed accelerometer structure. All dimensions in the following images are expressed in µm.

Isometric Drawing of Accelerometer Structure


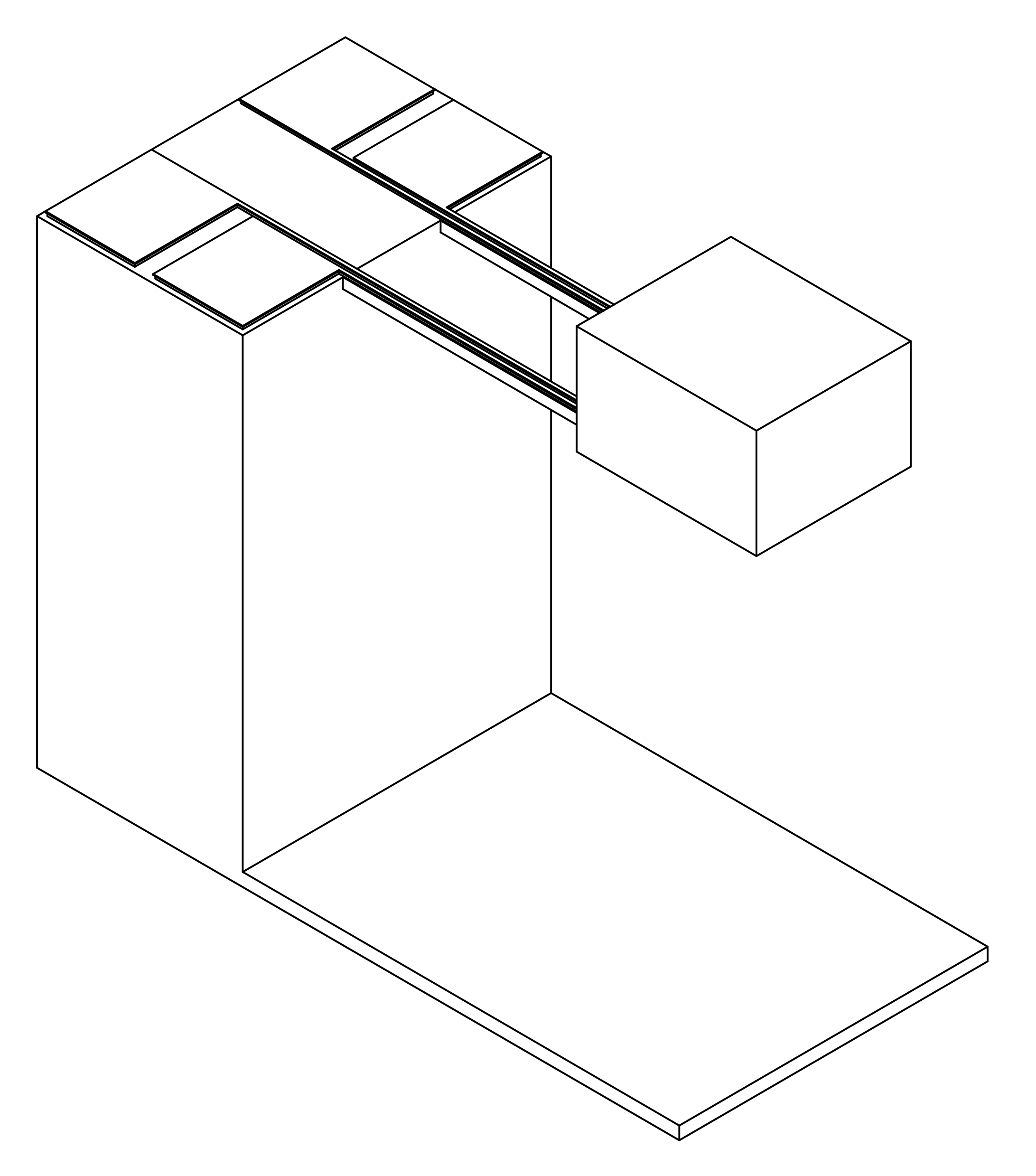


Side View Front View
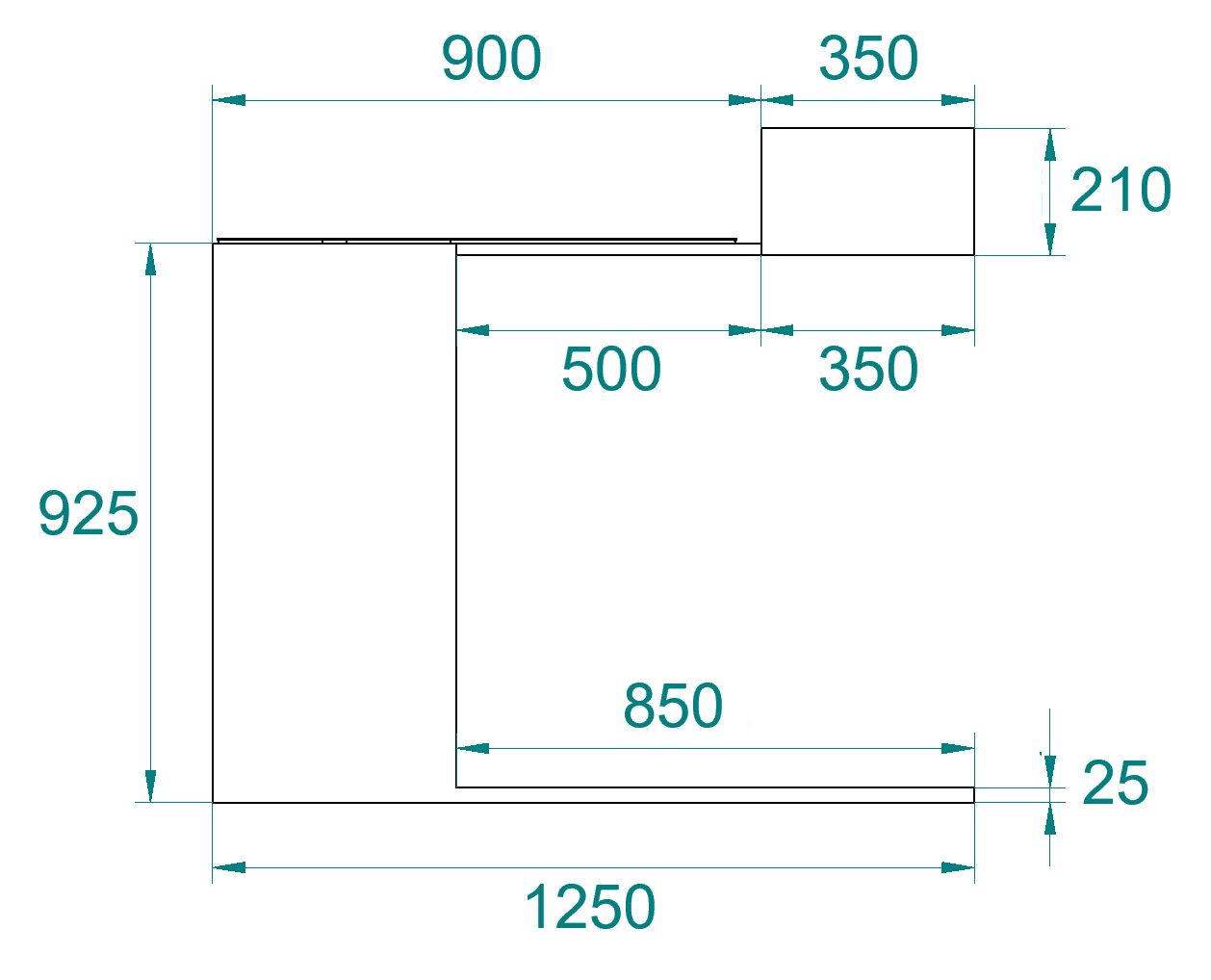

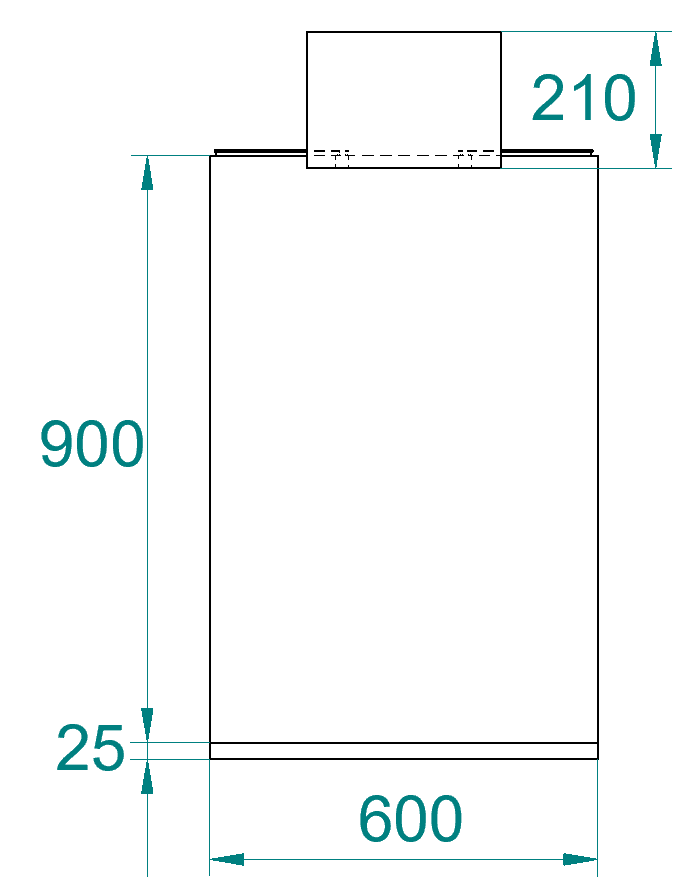


Top View


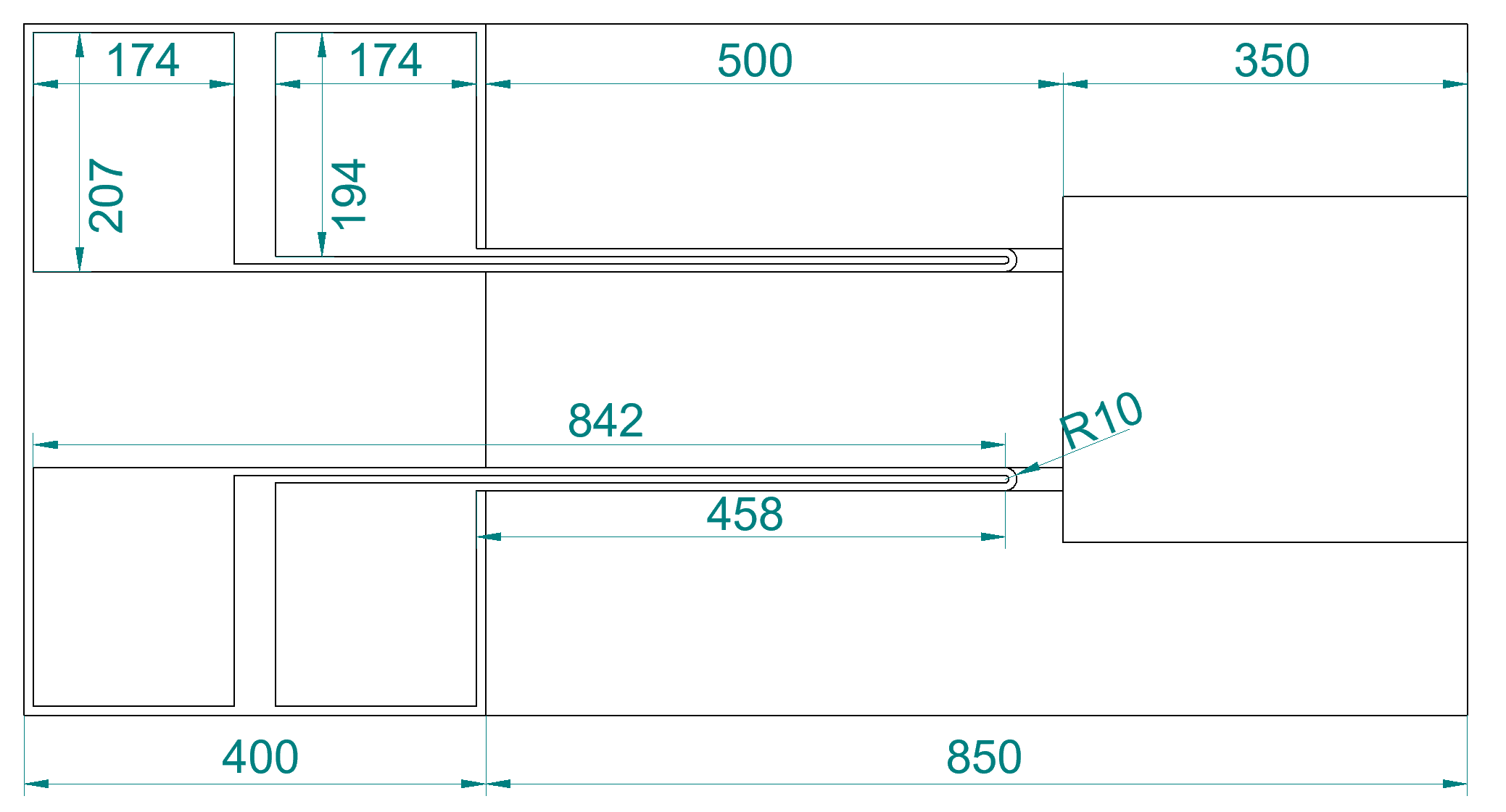


Cut-Out of the Front View


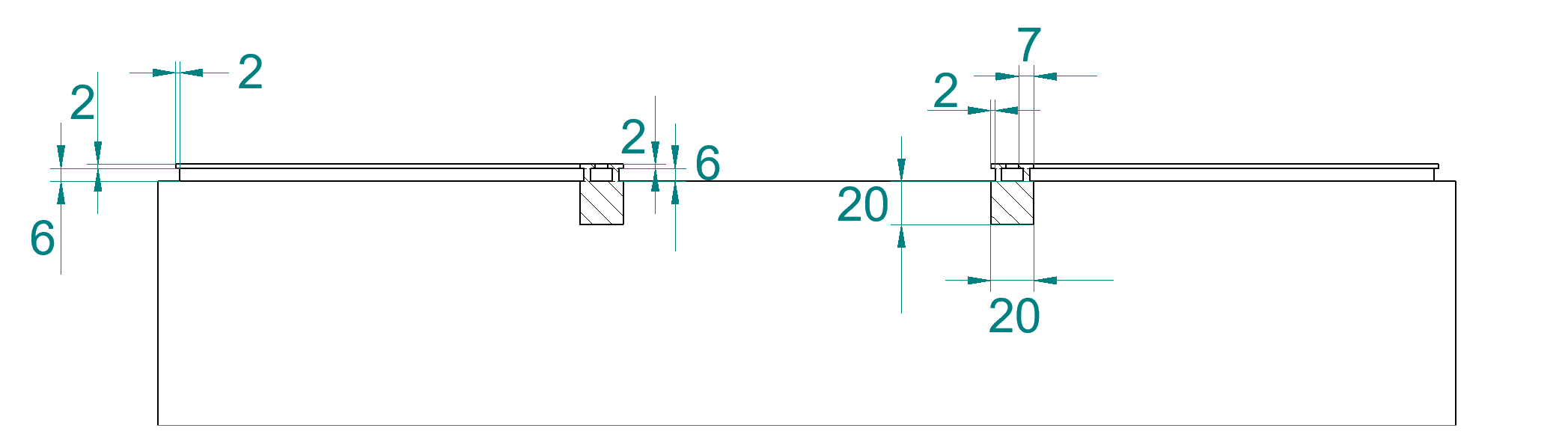


**S2. 3D Printing Parameters**

| **Writing Configuration**  GalvoScanMode ContinuousMode PiezoSettlingTime 10 GalvoAcceleration 10 StageVelocity 200  **Writing Parameters**  PowerScaling 1.0  **Shell Writing Parameters**  var **$shellLaserPower** = 100 var **$shellScanSpeed** = 100000  *Scaffold writing parameters* var **$scaffoldLaserPower** = 100 var **$scaffoldScanSpeed** = 100000  *Solid hatch lines writing parameters* var **$solidLaserPower** = 100 var **$solidScanSpeed** = 100000  *Base writing parameters* var **$baseLaserPower** = 100 var **$baseScanSpeed** = 100000  var **$interfacePos** = 0.5 | **Shell & Scaffold Printing**  ***Slicing*** *SlicingMode: Fixed* *Distance: 1* *SimplificationTolerance: 0.05* *FixSelfIntersections: on*  ***Shell*** *HatchingDistance: 0.5* *ContourCount: 22* *BaseSliceCount: 10* *Angle: auto* *ConcaveCornerMode: Sharp*  ***Scaffold*** *HatchingDistance: 0.5* *Type: Triangles* *WallSpacing: 20* *FloorSpacing: 25* *WallLineCount: 1* *FloorSliceCount: 1* *Offset X: 0 Y: 0 Z: 0* *Stagger: off*  **Solid Printing**  ***Slicing*** *SlicingMode: Fixed* *Distance: 1* *SimplificationTolerance: 0.05* *FixSelfIntersections: on*  ***Hatching*** *HatchingDistance: 0.5* *HatchingAngle: auto* |
| --- | --- |

**S3. Arrangement of Printing Blocks**


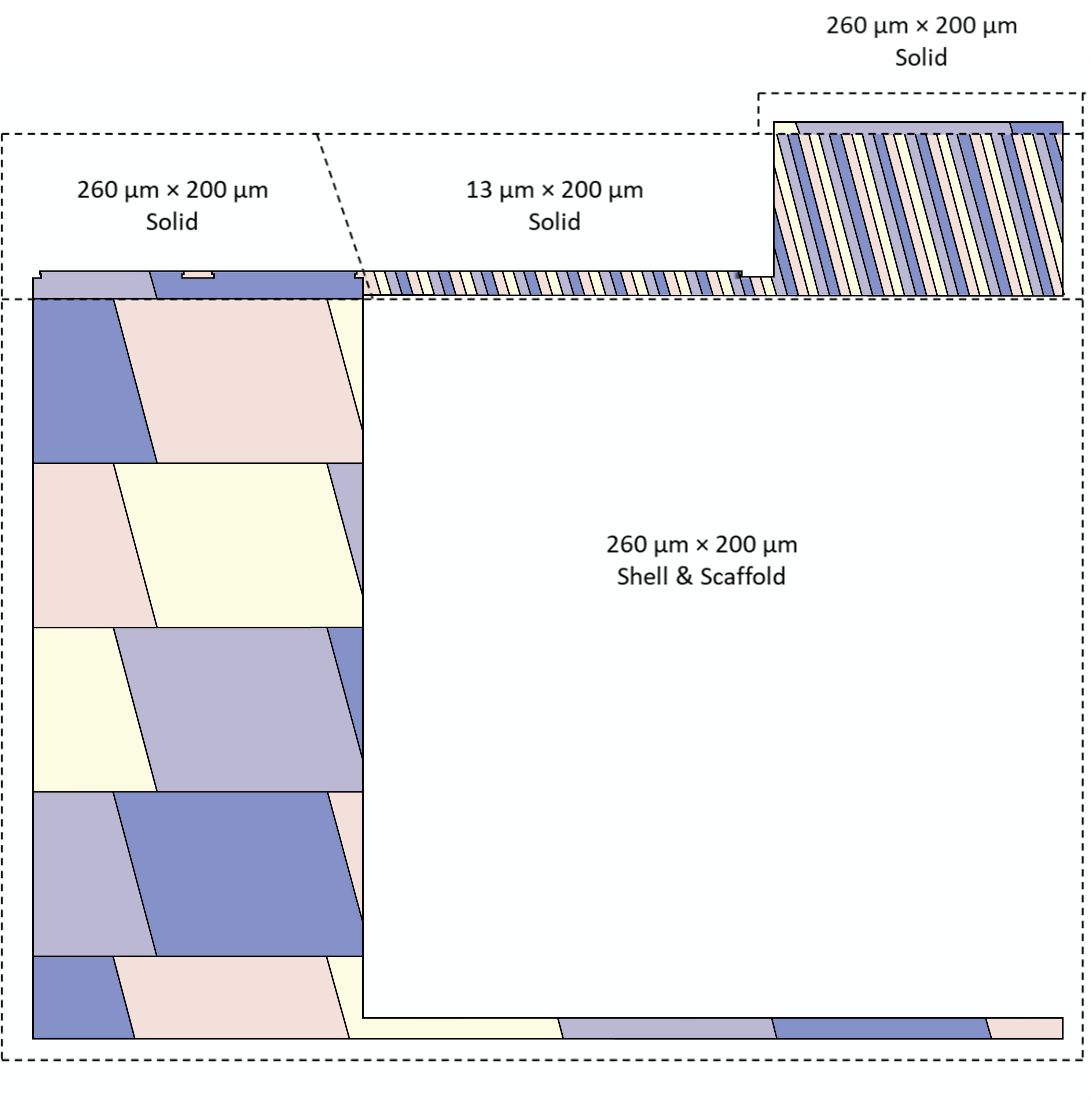


Figure S3: Side view of the 3D printed structure with the chosen stitching of the printing block arrangement. Large printing blocks have been used for the supporting pillar of the accelerometer structure, while short printing blocks have been used for the free-hanging parts (cantilever and proof mass). Block sizes (length x height) and printing mode are shown for each section of the structure.

**S4. SEM Images and Measured Dimensions of the 3D Printed Accelerometer**

| 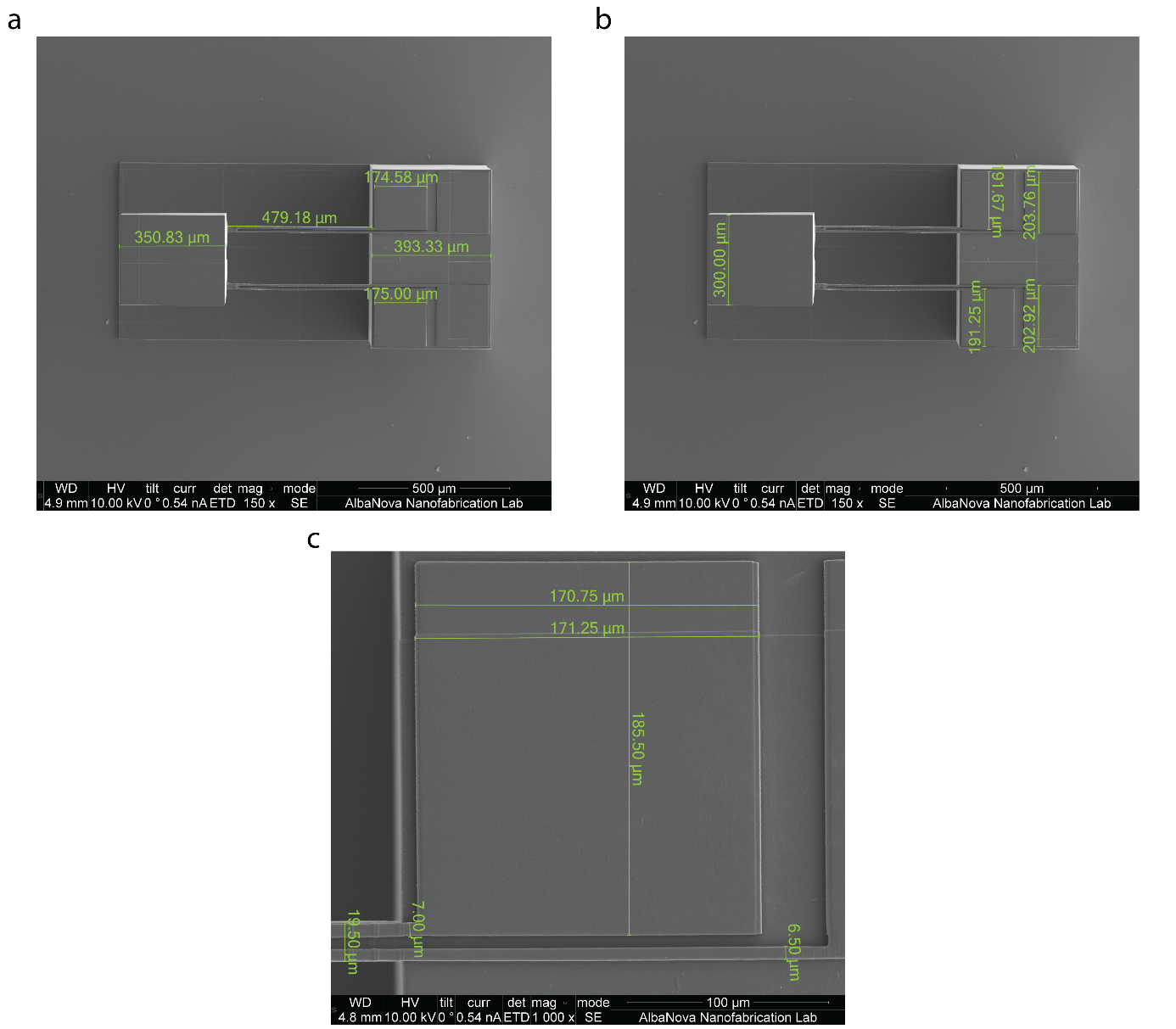 |
| --- |
| Figure S4: Top view with measured dimensions of the different parts of the 3D printed accelerometer structure: (a) length of the proof mass, the cantilevers, the electrodes and the supporting pillar. (b) Width of the proof mass and the electrodes. (c) Width and length of the electrodes, width of the cantilevers and of the top side of the strain gauge transducers. All accelerometer parts have maintained the nominal size, except the cantilevers, which feature an actual length of 480 µm instead of the nominal designed 500 µm. |

**S5. Theoretical analysis of cantilever bending**

According to the Euler-Bernoulli beam theory, bending of beams can be studied with the following set of differential equations:

$$EI\frac{d^{2}w(x)}{dx^{2}}=-M(x) (1)$$

$$\theta\left( x \right)=\frac{dw}{dx} (2)$$

where $E$ is the Young Modulus of the material of the cantilever, $I$ is the seconds moment of area (or area moment of inertia) of the vertical cross-section of the cantilever with respect to the neutral axis, $w(x)$ is the cantilever displacement in the $z$ direction along the $x$-axis, while $M(x)$ is the moment along the $x$-axis. The value of $E$ is extracted from the Comsol® simulation to be 6.5 GPa. The second moment of area for the cross-section of the beams is computed below on half beam with respect to the neutral axis, the position of which is calculated using the standard formulas. The half beam second moment of area is multiplied by two, to get the full beam moment, and then multiplied by two again to take into account the presence of two cantilevers.

| 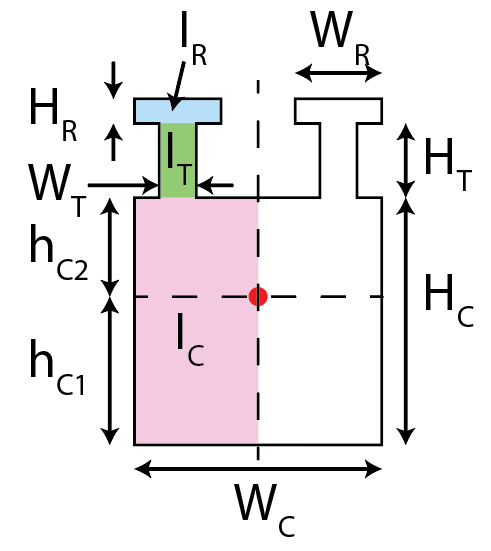 | $I_{C}=W_{C}(h_{C2}^{3}+h_{C1}^{3})/6 (3a)$  $I_{T}=W_{T}(\left( h_{C2}+H_{T} \right)^{3}-h_{C2}^{3})/3 (3b)$  $I_{R}=W_{r}(\left( h_{C2}+H_{T}+H_{R} \right)^{3}-\left( h_{C2}+H_{T} \right)^{3})/3 (3c)$  $I=2*2\left( I_{C}+I_{T}+I_{R} \right) (3d)$ |
| --- | --- |

A much simplified second moment of area could be computed, considering only the cross-section of the cantilever (the square $Hc*Wc$) but it would be about 51% of the value computed above, thus leading to a significant error.

To solve equations (1) and (2) we need to set both $M(x)$ and a coherent set of boundary conditions. Here, we developed two models. In the first model, the applied force is represented as a point force exerted on the axis passing through the center of mass of the proof mass and the mass is modeled as an infinitely stiff body. In the second model, pure bending is assumed, thus the load is modeled as a moment exerted at the end of the cantilever

**S5.1 Load as a point force**


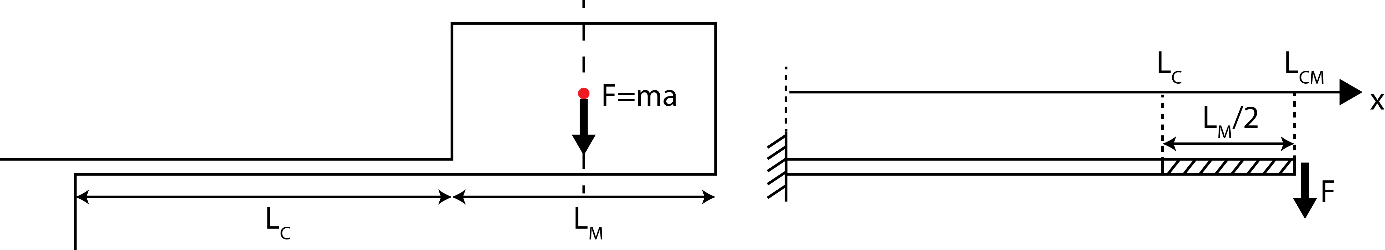


With a point force load along the vertical axis passing through the center of mass of the proof mass, momentum is represented by:

$$M\left( x \right)=F(L_{CM}-x) (4)$$

With this we compute the displacement and the slope at the end of the cantilever ($Lc$). To do so, we substitute (4) into (1) and integrate twice to obtain:

$$w\left( x \right)=\frac{Fx^{2}}{3EI}\left( 3L_{CM}-x \right) x<L_{C} (5)$$

$$\theta\left( x \right)=\frac{Fx}{EI}\left( L_{CM}-\frac{x}{2} \right) x<L_{C} (6)$$

From which we can compute displacement and slope at $L_{C}$ with $L_{CM}=L_{C}+L_{M}/2$

$$w\left( L_{C} \right)=\frac{FL_{C}^{3}}{3EI}\left( 1+\frac{3L_{M}}{4L_{C}} \right) (7)$$

$$\theta\left( L_{C} \right)=\frac{FL_{C}^{2}}{EI}\left( 1+\frac{L_{M}}{L_{C}} \right) (8)$$

Then, we estimate displacement at $L_{CM}$, considering the part of the cantilever between $L_{C}$ and $L_{CM}$ as a stiff body tilted with angle $\theta(L_{C})$ as :

$$w\left( L_{CM} \right)=w\left( L_{C} \right)+\theta\left( L_{C} \right)\frac{L_{M}}{2}=\frac{FL_{C}^{3}}{6EI}\left( 1+\frac{3L_{M}}{2L_{C}}+\frac{3L_{M}^{2}}{4L_{C}^{2}} \right) (9)$$

At this point, we compute the spring constant and resonance frequency:

$$k=\frac{F}{w\left( L_{CM} \right)}=\frac{6EI}{L_{C}^{3}\left( 1+\frac{3L_{M}}{2L_{C}}+\frac{3L_{M}^{2}}{4L_{C}^{2}} \right)} (10)$$

$$f_{res}=\frac{1}{2\pi}\sqrt{\left( \frac{k}{m} \right)}=\frac{1}{2\pi}\sqrt{\frac{6EI}{mL_{C}^{3}\left( 1+\frac{3L_{M}}{2L_{C}}+\frac{3L_{M}^{2}}{4L_{C}^{2}} \right)}}=1.89 kHz (11)$$

**S5.2 Load as a momentum**


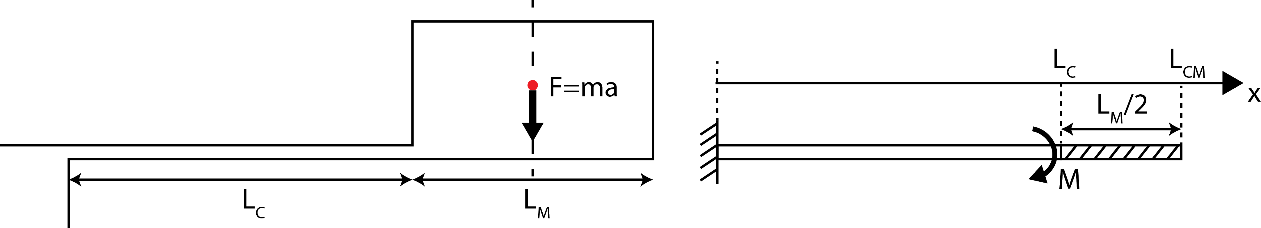


We assume a condition of pure bending, where the momentum is constant along the x-axis. We observe that the momentum at $x=0$ corresponds to $M=F(L_{c}+L_{M}/2)$. We substitute this value into (1) and integrate twice to obtain:

$$w\left( x \right)=\frac{Mx^{2}}{2EI} x<L_{C} (12)$$

$$\theta\left( x \right)=\frac{Mx}{EI} x<L_{C} (13)$$

Same as in the previous section, we compute displacement and slope at $L_{C}$, then we estimate the displacement at $L_{CM}$:

$$w\left( L_{CM} \right)=w\left( L_{C} \right)+\theta\left( L_{C} \right)\frac{L_{M}}{2}=\frac{ML_{C}}{2EI}\left( L_{C}+L_{M} \right)=\frac{FL_{C}^{3}}{2EI}\left( 1+\frac{L_{M}}{2L_{C}} \right)\left( 1+\frac{L_{M}}{L_{C}} \right) (14)$$

Then, we compute the spring constant and the resonance frequency:

$$k=\frac{F}{w\left( L_{CM} \right)}=\frac{2EI}{L_{C}^{3}\left( 1+\frac{L_{M}}{2L_{C}} \right)\left( 1+\frac{L_{M}}{L_{C}} \right)} (15)$$

$$f_{res}=\frac{1}{2\pi}\sqrt{\left( \frac{k}{m} \right)}=\frac{1}{2\pi}\sqrt{\frac{6EI}{mL_{C}^{3} \left( 1+\frac{L_{M}}{2L_{C}} \right)\left( 1+\frac{L_{M}}{L_{C}} \right)}}=1.58 kHz (16)$$

**S5.3 Responsivity**

The responsivity of the accelerometer is defined as $\frac{\Delta R/R}{a}$ and can be estimated considering the definition of the gauge-factor and the relationship between the bending of the cantilever and the strain in the resistor [37]:

$GF= \frac{\Delta R/R}{\varepsilon} (17)$

$$\varepsilon=\frac{3\left( 1-\frac{L_{r}}{2L_{C}} \right)z_{n}}{L_{C}^{2}}w\left( L_{C} \right) (18)$$

where $\varepsilon$ is the strain applied to the resistor, $L_{r}$ is the length of the resistor and $z_{n}$ is the vertical distance between the resistor and the neutral plane of the cantilever.

The responsivity of the accelerometer with the point force model can then be computed by substituting Eq. (7) into Eq. (18), and then Eq. (18) into Eq. (17):

$$R_{pf}=\frac{GF\varepsilon}{a}=GF\frac{3\left( 1-\frac{L_{r}}{2L_{C}} \right)z_{n}}{L_{C}^{2}}\frac{L_{C}^{3}}{3EI}\left( 1+\frac{3L_{M}}{4L_{C}} \right)m=\frac{GF}{E}\frac{z_{n}L_{C}}{I}\left( 1-\frac{L_{r}}{2L_{C}} \right) \left( 1+\frac{3L_{M}}{4L_{C}} \right)m (19)$$

In the same way, the responsivity with an applied momentum is computed to be:

$$R_{m}= GF\frac{3\left( 1-\frac{L_{r}}{2L_{C}} \right)z_{n}}{L_{C}^{2}}\frac{L_{c}^{3}}{2EI}\left( L_{C}+\frac{L_{M}}{2} \right)m=\frac{GF}{E}\frac{z_{n}L_{C}}{I}\left( 1-\frac{L_{r}}{2L_{C}} \right)\frac{3}{2}\left( 1+\frac{L_{M}}{2L_{C}} \right)m (20)$$

**S6. Measurements of Amplitude of Oscillation of the Proof Mass**


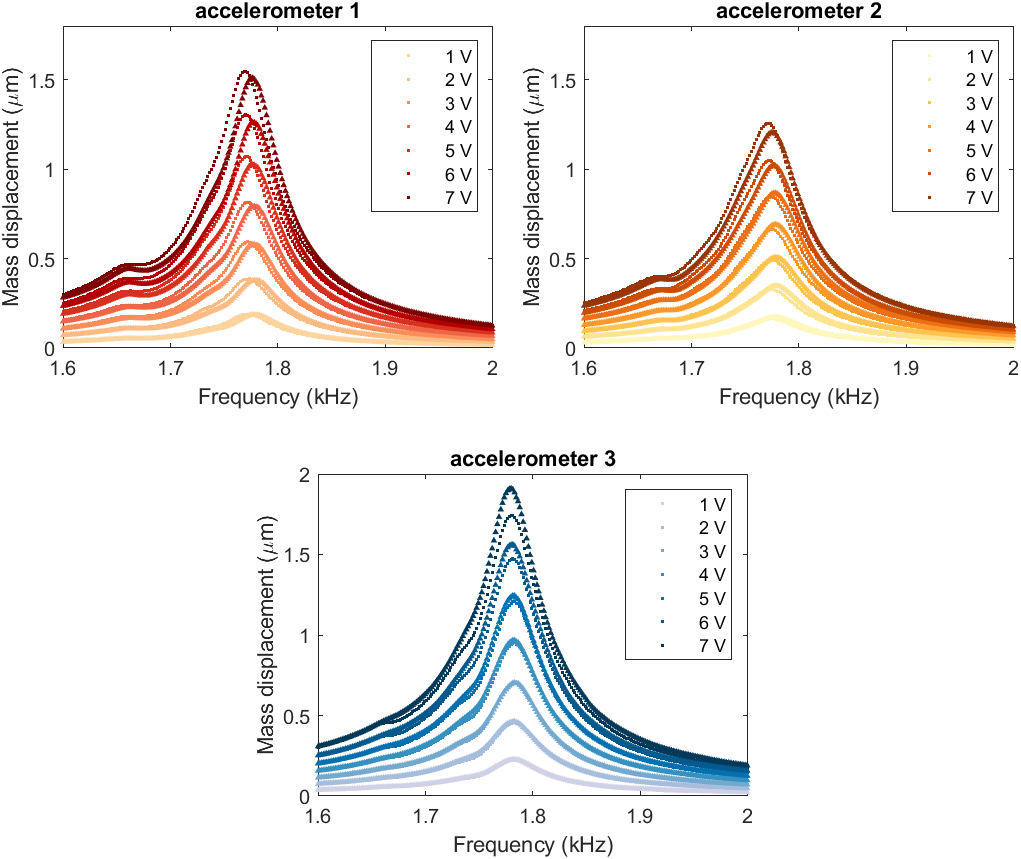


Figure S6: Amplitude of oscillations of the proof mass measured by the Laser Doppler Vibrometer at different oscillation frequencies (plotted dots). Each color corresponds to a different driving voltage applied to the piezoshaker. Two sweeps have been performed at each driving voltage. We estimated the Q factor with a Lorentzian fit. The estimated values of mean resonance frequency, Q factor and the maximum amplitude of oscillation for each driving voltage are reported in the following table.
R^2^ > 0.98 for all fits.

|  | Accelerometer 1 | | | Accelerometer 2 | | | Accelerometer 3 | | |
| --- | --- | --- | --- | --- | --- | --- | --- | --- | --- |
| Driving Voltage (V_rms_) | $f_{res}$  (kHz) | Q-Factor | Mass disp. (µm) | $f_{res}$  (kHz) | Q-Factor | Mass disp. (µm) | $f_{res}$  (kHz) | Q-Factor | Mass disp. (µm) |
| 1 | 1.775 | 37 | 0.19 | 1.776 | 34.1 | 0.17 | 1.782 | 36.5 | 0.23 |
| 2 | 1.775 | 37 | 0.38 | 1.776 | 34 | 0.34 | 1.782 | 36.5 | 0.47 |
| 3 | 1.774 | 37.2 | 0.59 | 1.776 | 33.3 | 0.49 | 1.782 | 36.7 | 0.73 |
| 4 | 1.774 | 37 | 0.80 | 1.776 | 32.9 | 0.68 | 1.782 | 36.3 | 0.99 |
| 5 | 1.773 | 36.3 | 1.05 | 1.775 | 32.6 | 0.85 | 1.782 | 36.4 | 1.27 |
| 6 | 1.773 | 36.9 | 1.28 | 1.774 | 32.3 | 1.03 | 1.781 | 36.1 | 1.58 |
| 7 | 1.773 | 36.6 | 1.53 | 1.773 | 31.8 | 1.23 | 1.780 | 35.8 | 1.89 |

**S7. Environmental measurements during tests of long-term stability**


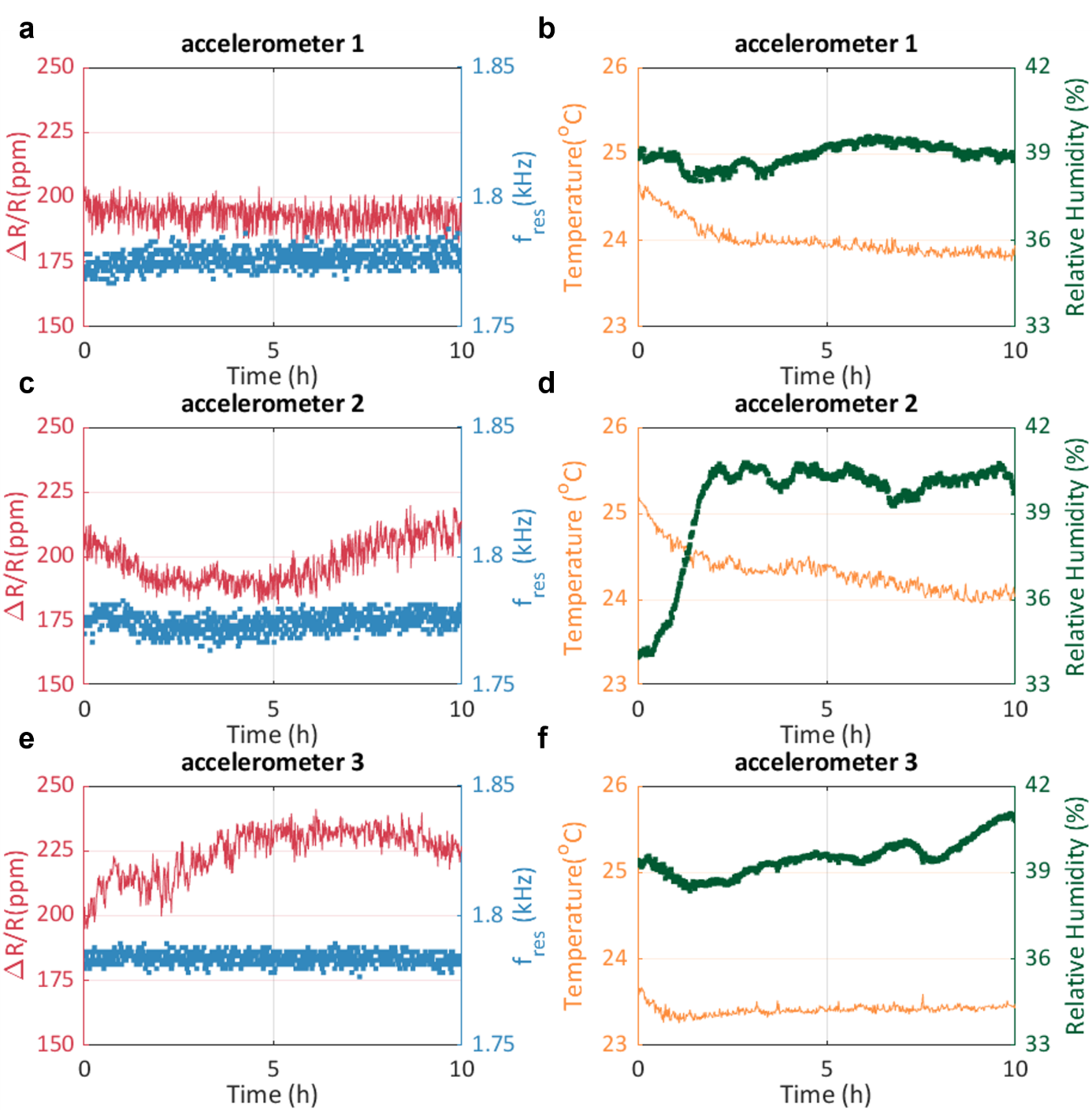


Figure S7: long-term stability measurements of ΔR/R and resonance frequency (a-c-e) and measurements of the temperature and relative humidity during those experiments (b-d-f) are plotted side-by-side for the three different accelerometers. Temperature and relative humidity were measured with a sensor SHT40 (Sensirion AG, Switzerland) placed on the optical table, next to the mechanical stages where the piezoshaker and the devices were positioned. For all experiments, the temperature oscillated within a range of 24 °C ± 0.8 °C, while the relative humidity varied in a range of 39 % ± 1.5 %. We computed the Pearsons correlation coefficients between each environmental parameters (temperature and humidity) and each performance parameter (ΔR/R and resonance frequency). All the computed coefficients were lower than 0.4 and, in many cases with opposite signs between different devices. This shows that the very small variations of environmental parameters did not significantly affect the measurements of the accelerometer stability.

**S8. Simulations of Responsivities and Cross-Responsivities**

To perform the simulations of cross-responsivities we built the full geometry of the device in the COMSOL® model, because Y-axis accelerations would break the symmetry of the model previously built and described in the Materials and methods.

We have run three separate simulations, applying to the full structure sweeps of acceleration from 0 to 1 g with steps of 0.2 g in the 3 different directions and extracted the computed resistance for each acceleration applied. The gauge-factor of the resistive material was not adapted to the value we computed and reported in the Discussion section of the paper, because it affects all the responsivities in the different directions in the same way, and thus it does not affect the cross-responsivities. The computed values of relative resistance change ΔR/R are reported in the table below, together with the cross-responsivity of accelerations along the x- ad y-axis with respect to the z-axis.

| Acceleration(g) | ΔR/R (ppm) – x | ΔR/R (ppm)- y | ΔR/R (ppm) - z |
| --- | --- | --- | --- |
| 0 | 0 | 0 | 0 |
| 0.2 | 0.31 | 0.019 | 1.43 |
| 0.4 | 0.62 | 0.042 | 2.87 |
| 0.6 | 0.94 | 0.064 | 4.31 |
| 0.8 | 1.25 | 0.087 | 5.75 |
| 1 | 1.57 | 0.11 | 7.18 |
| Cross-responsivity | 0.218 | 0.015 | / |

From the numbers, we can see a cross-responsivity of about 22% between the x- and the z-axis, and of 1.5% between the y- and the z-axis. Our device was not designed to minimize cross-responsivity, thus the cross-responsivity along the x-axis is not negligible. However, here we propose a simple modification of our accelerometer design, aimed at minimizing cross-responsivity along the x-axis. The large cross-responsivity originates from the large distance of the center of mass of the proof-mass from the neutral axis of the cantilevers, which results in a large moment applied to the cantilevers when an acceleration on the x-axis is applied. To avoid such large cross-responsivity, it is possible to fabricate a device with a proof mass with center of mass aligned to the neutral axis of the cantilevers, as shown in Figure S8. By doing so, the cross-responsivity between the x- and the z-axis are dramatically reduced to 0.4%, without affecting the responsivity along the z-axis and only slightly increasing the y-axis responsivity to 3.6%.

| 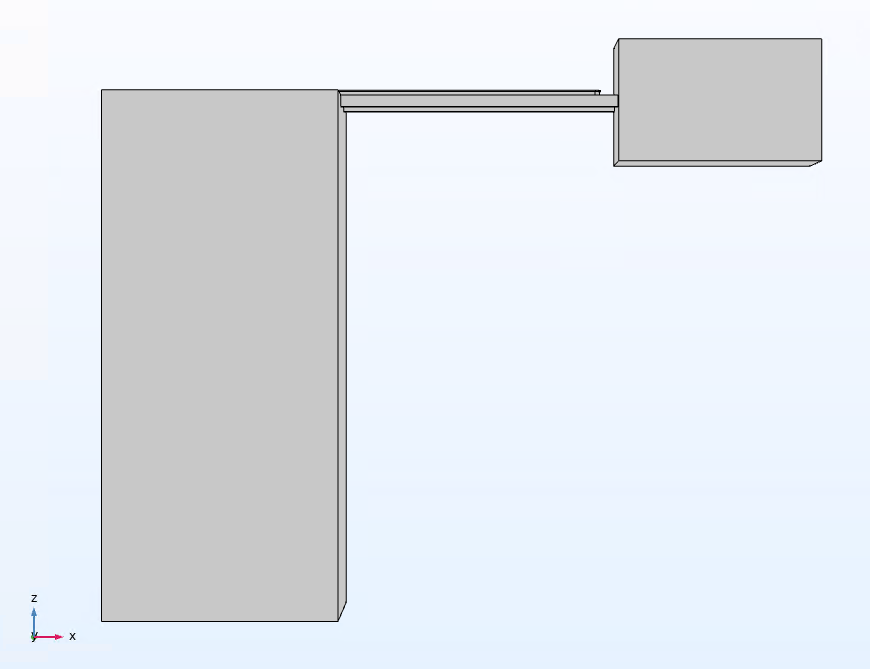 | \| Acc.(g) \| ΔR/R (ppm) – x \| ΔR/R (ppm)- y \| ΔR/R (ppm) - z \| \| --- \| --- \| --- \| --- \| \| 0 \| 0 \| 0 \| 0 \| \| 0.2 \| 0.007 \| 0.055 \| 1.43 \| \| 0.4 \| 0.012 \| 0.107 \| 2.87 \| \| 0.6 \| 0.018 \| 0.16 \| 4.31 \| \| 0.8 \| 0.23 \| 0.21 \| 5.75 \| \| 1 \| 0.28 \| 0.26 \| 7.18 \| \| Cross-responsivity \| 0.0038 \| 0.036 \| / \| |
| --- | --- | --- | --- | --- | --- | --- | --- | --- | --- | --- | --- | --- | --- | --- | --- | --- | --- | --- | --- | --- | --- | --- | --- | --- | --- | --- | --- | --- | --- | --- | --- | --- | --- |
| Figure S8.1: alternative accelerometer design to minimize cross-responsivity along the x-axis. |  |

We exploited the 3D printing capabilities of two-photon polymerization to fabricate an accelerometer structure with the design shown in Figure S8.1. To ensure successful printing of this structure, we divided the mass horizontally in three different volumes and fabricated it in three stages. First, the support structure and the cantilevers of the accelerometer were printed. Thereafter, starting from the bottom end of the cantilevers, the central part of the proof mass with a thickness of 50 µm was printed using 14 µm long printing blocks. Next, the bottom part of the proof mass was printed proceeding in the opposite direction, starting from the already printed layer and going downwards. Finally, the top part of the proof mass was printed on top of the already printed structure. The print layout and an SEM image of the successfully printed accelerometer structure are shown in Figure S8.2.


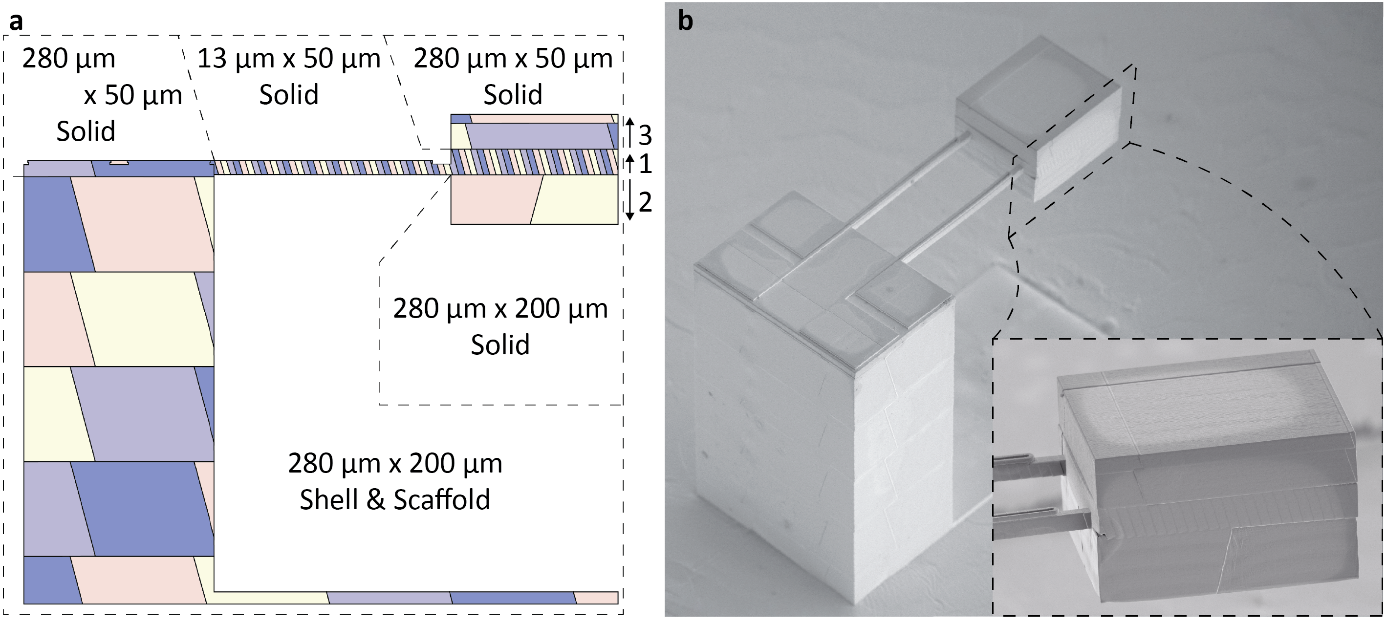


Figure 8.2: 3D printed accelerometer structure with low cross-responsivity design. (a) Printing block arrangement with specified printing direction and printing order of the printing blocks in the proof mass. (b) SEM image of the printed accelerometer structure with zoomed-in proof mass and its connection to the cantilevers in the inset.
